# Performance evaluation of the Panbio COVID-19/Flu A&B Panel for detection of SARS-CoV-2, influenza A, and influenza B antigens using mid-turbinate nasal swabs

**DOI:** 10.1128/jcm.00207-24

**Published:** 2024-06-18

**Authors:** Shao-Hua Yu, Keun-Ju Kim, Chien-Chang Lee, Yanely Pineiro Puebla, Gelza Mae A. Zabat, Hong-Mo Shih, Po-Ren Hsueh

**Affiliations:** 1 Department of Emergency Medicine, China Medical University Hospital, China Medical University, Taichung, Taiwan; 2 Department of Laboratory Medicine, Korea University College of Medicine, Seoul, South Korea; 3 Department of Emergency Medicine, National Taiwan University, Taipei, Taiwan; 4 Palm Springs Community Health Center, Palm Springs, California, USA; 5 Health Cube Medical Clinics, Philippines, Philippines; 6 Department of Laboratory Medicine, China Medical University Hospital, School of Medicine, China Medical University, Taichung, Taiwan; 7 Division of Infectious Diseases, Department of Internal Medicine, China Medical University Hospital, School of Medicine, China Medical University, Taichung, Taiwan; Wadsworth Center - NYSDOH, Albany, New York, USA

**Keywords:** COVID-19, SARS-CoV-2, Panbio COVID-19/Flu A&B Panel, cobas SARS-CoV-2 & Influenza A/B qualitative assay, sensitivity, specificity

## Abstract

The Panbio COVID-19/Flu A&B Panel (Abbott) is an *in vitro* diagnostic rapid test designed for the qualitative detection of nucleocapsid proteins SARS-CoV-2 and nucleoprotein influenza A and B antigens in nasal mid-turbinate (NMT) swab specimens from symptomatic individuals meeting COVID-19 and influenza clinical and/or epidemiological criteria. This study, the largest global one to date using fresh samples, aimed to assess the diagnostic sensitivity and specificity of the Panbio COVID-19/Flu A&B Panel in freshly collected NMT swab specimens from individuals suspected of respiratory viral infection consistent with COVID-19 and/or influenza within the first 5 days of symptom onset compared with results obtained with the cobas SARS-CoV-2 and influenza A/B qualitative assay (cobas 6800/8800 systems), which were tested using nasopharyngeal swab samples. A total of 512 evaluable subjects were enrolled in the COVID-19 cohort across 18 sites, and 1,148 evaluable subjects were enrolled in the influenza cohort across 22 sites in the Asia-Pacific, Europe, and the USA. The Panbio COVID-19/Flu A&B Panel demonstrated a sensitivity of 80.4% and a specificity of 99.7% for COVID-19. For influenza A, the sensitivity and specificity rates were 80.6% and 99.3%, respectively. Likewise, for influenza B, the sensitivity and specificity rates were 80.8% and 99.4%, respectively. In conclusion, the Panbio COVID-19/Flu A&B Panel emerges as a suitable rapid test for detecting COVID-19 and influenza in symptomatic subjects across diverse global populations, exhibiting high sensitivity. The assay achieved a sensitivity of 94.4% in samples with Ct ≤24 for COVID-19 and 92.6% in samples with Ct ≤30 for influenza A and B.

**IMPORTANCE:**

The Panbio COVID-19/Flu A&B Panel is a suitable rapid test for detecting COVID-19 and influenza in symptomatic subjects across diverse global populations, exhibiting high sensitivity. The assay achieved a sensitivity of 94.0% in samples with Ct ≤24 for COVID-19 and 92.6% in samples with Ct ≤30 for influenza A and B.

## INTRODUCTION

Human coronaviruses (HCoVs) cause various respiratory conditions, such as the common cold, bronchiolitis, and pneumonia (1). Infections from HCoVs progress rapidly due to increased proliferation rates via nucleotide substitution and recombination (2). Since the emergence of a new homologous strain of coronavirus (CoV-2) in December 2019 in China, identified as the severe acute respiratory syndrome coronavirus 2 (SARS-CoV-2), more than 700 million confirmed cases of coronavirus disease 2019 (COVID-19) have been reported, resulting in over 7 million deaths globally (3). The pandemic has seen multiple waves with different variants (4). Early diagnosis is critical for the treatment of COVID-19. At present, most reported cases have been identified through qualitative detection methods of SARS-CoV-2 RNA, antigens, or antibodies (5).

Influenza viruses, members of the Orthomyxoviridae family, are negative-sense single-stranded RNA viruses. Influenza A and B cause significant morbidity and mortality across all age groups, leading to local outbreaks and seasonal epidemics. These infections are responsible for approximately 500,000 deaths annually and infect up to a quarter of the global human population (6). Symptoms range from the common cold to lethal pneumonia or multi-organ failure (7). Rapid diagnosis of influenza A and B is crucial due to the availability of effective antiviral therapy, which reduces hospital stays and healthcare costs, as well as deceases the inappropriate use of antibiotics (8).

The evidence of the ongoing COVID-19 pandemic is expected to continue posing a critical healthcare concern in the coming years. Healthcare systems may face overlapping outbreaks of SARS-CoV-2 and influenza. Rapid diagnostic tools capable of differentiating between COVID-19 and influenza are essential for accurate diagnosis, care, and treatment (9, 10). Utilizing point-of-care tests can also help in avoiding the inappropriate use of antibiotics (11–14). Self-testing for detecting SARS-CoV-2 and influenza A and B viruses enables individuals to self-diagnose and respond more swiftly to the disease, a practice strongly recommended by the World Health Organization (WHO) (15).

This study aims to assess the performance of the Panbio COVID-19/Flu A&B Panel (Abbott, Abbott Park, IL, USA) for detecting SARS-CoV-2 and influenza A and B antigens compared with results by the cobas SARS-CoV-2 and influenza A/B qualitative assay (cobas assay) (Roche Molecular Systems, Inc.*,* Branchburg*,* NJ*,* USA) by using nasal samples. The claimed positive percent agreement (PPA) of the cobas assay was 96.4%, 100.0%, and 100.0% for SARS-CoV-2, influenza A, and influenza B, respectively, evaluated using nasopharyngeal specimens (16). The primary endpoint of this study is to evaluate the clinical performance (diagnostic sensitivity/diagnostic specificity) of the Panbio COVID-19/Flu A&B Panel using freshly collected nasal mid-turbinate (NMT) swab specimens from individuals suspected of respiratory viral infection consistent with COVID-19 and/or influenza by their healthcare provider within the first 5 days of the onset of symptoms in a near patient testing setting compared with influenza and SARS-CoV-2 RT-PCR tested using nasopharyngeal (NP) swab samples. NP swabs are considered the gold standard for the collection of SARS-CoV-2 and influenza samples (17, 18). Therefore, this study utilized the most stringent method comparison by evaluating the sensitivity of the Panbio COVID-19/Flu A&B Panel using NMT swab specimens to a PCR method that utilized NP swab specimens.

## MATERIALS AND METHODS

### Panbio COVID-19/Flu A&B Panel

The Panbio COVID-19/Flu A&B Panel is an *in vitro* diagnostic rapid test for the qualitative detection of nucleocapsid protein SARS-CoV-2 and nucleoprotein influenza A and B antigens in human NMT swab specimens from symptomatic individuals meeting COVID-19 and influenza clinical and/or epidemiological criteria. This test is intended for use by trained professionals in a laboratory and near-patient setting as an aid in the diagnosis of SARS-CoV-2 and influenza infection.

### Cobas SARS-CoV-2 and influenza A/B assay (cobas assay)

The cobas assay is conducted on the cobas 6800 for reference testing in China and the cobas 8800 for reference testing in the UK for samples collected outside China. It is a qualitative test that detects and differentiates SARS-CoV-2, influenza A, and influenza B virus RNA. In this study, 200 µL of viral transport medium from a NP swab specimen was utilized for each analysis. The cobas assay designated a specimen as positive if either or both target genes (ORF1a/b and N genes) of SARS-CoV-2 were detected.

### Subjects

In the COVID-19 cohort, subjects were enrolled at 18 sites across six countries in Asia-Pacific (APAC), Europe (EU), and the United States (US) (Table 1). In the influenza cohort, subjects were enrolled at 22 sites across seven countries (Table 1). Male and female individuals of diverse ethnicities aged ≥1 year were recruited. Subjects from all age groups who met the following criteria were prospectively enrolled: (1) able and willing to provide written informed consent or assent (2), suspected of having a respiratory viral infection consistent with COVID-19 and/or influenza by their healthcare provider within the first 5 days of the onset of symptoms, and (3) presenting at least two of the following symptoms: fever, headache, tiredness, dry cough, sore throat, runny or stuffy nose, muscle aches, loss of smell, loss of taste or shortness of breath.

**TABLE 1:** Number of participating countries and sites and number of evaluable subjects enrolled in this study

Country	No. of participating sites		No. of evaluable subjects enrolled	
	COVID-19	Influenza A and B	COVID-19	Influenza A and B
China	0	2	0	90
Georgia	6	7	116	345
Philippines	2	3	34	74
Poland	1	1	59	170
South Korea	2	2	20	31
Taiwan	3	3	157	281
United States	4	4	126	157
Total	18	22	512	1,148

Subjects were excluded from the study if they (1) had a nasal or a NP swab taken within the previous 4 h and were not available for further testing after 4 h had elapsed (2), had active nose bleeds or acute facial injuries/trauma (3), received a nasal vaccine (i.e., FluMist) within the previous 14 days (4), were taking or had taken an antiviral medication—e.g., amantadine, rimantadine, zanamivir, oseltamivir phosphate, and rimantadine—for influenza or COVID-19 within the previous 30 days (5), were enrolled in a study to evaluate an investigational drug (6), were unwilling or unable to provide informed consent, or (7) were part of a vulnerable population as deemed inappropriate for study by the site’s principal investigator and/or reviewing ethic committee. After verifying that each participant met all study inclusion criteria and none of the exclusion criteria, the study staff obtained written informed consent. Subject demographics and a brief medical history were collected.

### Sample collection and testing

**Fig 1:**
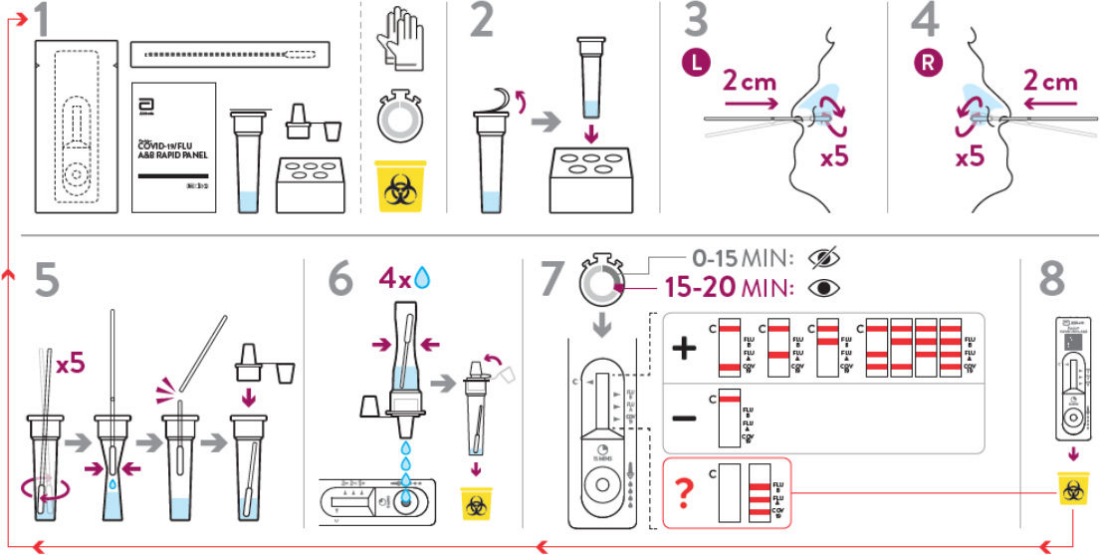
Instruction for the Panbio COVID-19/Flu A&B panel.

The infection status of subjects was unknown to subjects or study staff members before testing. Two samples were collected from each subject. One nasal mid-turbinate (NMT) swab sample was collected from both nostrils by a trained healthcare worker and tested according to the instructions for use (IFU) of the Panbio COVID-19/Flu A&B Panel (Fig. 1). The sample was immediately used for testing with the Panbio COVID-19/Flu A&B Panel.

In addition, one nasopharyngeal sample was collected from one nostril by a trained healthcare worker, eluted in universal transport media (UTM) and stored frozen. The sample was transported while frozen to the central laboratory for RT-PCR assay. Left-over UTM was stored at −80°C. The test results obtained using the Panbio COVID-19/Flu A&B Panel were compared with those obtained using the NP swab samples taken from the same subject and tested by the RT-PCR assay.

### Control testing

When using the Panbio COVID-19/Flu A&B Panel, the sites performed control testing using the external positive control swabs available in each Panbio COVID-19/Flu A&B Panel control kit. Controls were tested according to the instructions provided in the product’s IFU (19).

### Data collection

At each site, study data were entered into an Electronic Data Capture (EDC) System. The data recorded in the database was source-verified from study-related source documents to ensure that all data were complete, accurate, and consistent with source documentation.

### Statistical analysis

The sensitivity and specificity of the Panbio COVID-19/Flu A&B Panel using NMT swabs were evaluated against SARS-CoV-2, influenza A, and influenza B RT-PCR results using NP swabs. Subgroup analyses were conducted on subjects stratified by days since symptom onset, infectivity, and cycle threshold (Ct) value by RT-PCR. Additional stratifications were also performed as follows: (1) age (≤13, 14–23, 24–64, and ≥65 years) (2), pediatric/adult or binary age range (<18, ≥18 years) (3), regions (Asia, Europe, and the USA), and (4) vaccination status (vaccinated and not vaccinated).

The data analysis was comprised of point estimation and 95% confidence intervals of sensitivity and specificity for each target disease. A positive test result for any analyte could be considered as true positive for any of the three pathogens. Thus, analysis was comprised of an “any pathogen” sensitivity assessment in addition to the “correct pathogen” sensitivity that would support the use of the device to discriminate between the three pathogens. Subgroup analysis among the four main arms (positive for either of the three pathogens and negative for all of them) was performed.

## RESULTS

**Fig 2:**
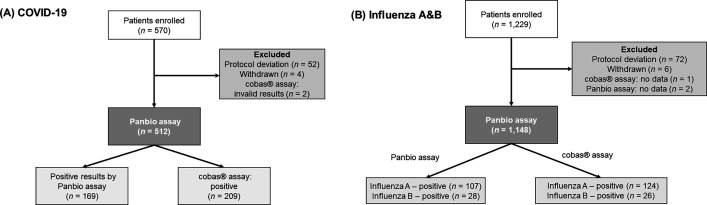
Flow chart of patient enrollment for (**A**) COVID-19 and (**B**) influenza A and B.

An evaluation of the Panbio COVID-19/Flu A&B Panel was performed from December 2022 to March 2023. In the COVID-19 cohort, 570 subjects were initially enrolled, with 512 subjects considered evaluable (Fig. 2A). These individuals were recruited from 18 sites across six countries and three continents (Table 1). The evaluable subjects in the COVID-19 cohort ranged in age from 1 to 88 years old, with a median age of 39 years. Among them, 300 (58.6%) were female (including six who are pregnant, 1.2%) and 212 (41.4%) were male. Additionally, 494 subjects were adults (≥18 years old), and 18 subjects were minors (<18 years old). Reported ethnicities included Black (*n* = 14, 2.7%), Chinese (*n* = 154, 30.1%), Filipino (34, 6.6%), Indian (*n* = 1, 0.2%), Japanese (*n* = 1, 0.2%), Korean (*n* = 20, 3.9%), Vietnamese (*n* = 2, 0.4%), White (*n* = 276, 53.9%), and others (*n* = 10, 2.0%). Within this cohort, 361 subjects (70.5%) had received various doses of COVID-19 vaccines, whereas 77 (15.0%) had received the influenza vaccine. All 512 (100.0%) subjects reported COVID-19 symptoms, with a median of 3.0 days (range, 1.0–5.0 days) since the onset of symptoms.

The influenza cohort initially had 1,229 enrolled subjects, of whom 1,148 subjects were evaluable (Fig. 2B). These individuals were recruited from 22 sites across seven countries and three continents (Table 1). The evaluable subjects in the influenza cohort ranged in age from 1 to 90 years, with a median age of 36 years. Among them, 682 (59.4%) were female (including seven who are pregnant, 0.6%), and 466 (40.6%) were male. Additionally, 1,016 subjects were adults, and 132 subjects were minors. Reported ethnicities included Black (*n* = 20, 1.7%), Chinese (*n* = 368, 32.1%), Filipino (*n* = 74, 6.4%), Indian (*n* = 2, 0.2%), Japanese (*n* = 1, 0.1%), Korean (*n* = 32, 2.8%), Vietnamese (*n* = 2, 0.2%), White (*n* = 631, 55.0%), and others (*n* = 18, 1.6%). Within this cohort, 827 subjects (72.0%) and 150 (13.1%) had received COVID-19 and influenza vaccines, respectively. All 1,148 (100.0%) subjects reported influenza symptoms, with a median of 2 days (range, 1–5 days) since the onset of symptoms.

**Fig 3:**
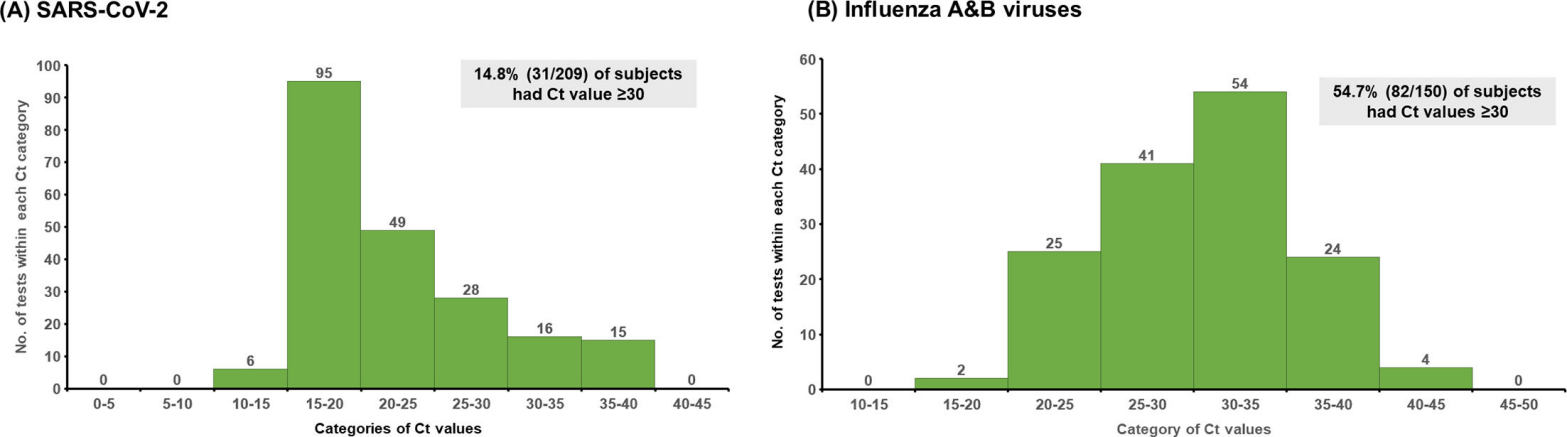
Distribution of cycle threshold (Ct) values for (**A**) SARS-CoV-2 and (**B**) influenza A and B viruses using cobas SARS-CoV-2 and influenza A/B qualitative assay.

In the overall data set, the percentage of tested positive subjects with Ct value ≥30 was 14.8% within the COVID-19 cohort, as illustrated in Fig. 3A. Within the influenza group, this percentage was 54.7%, as depicted in Fig. 3B.

**Fig 4:**
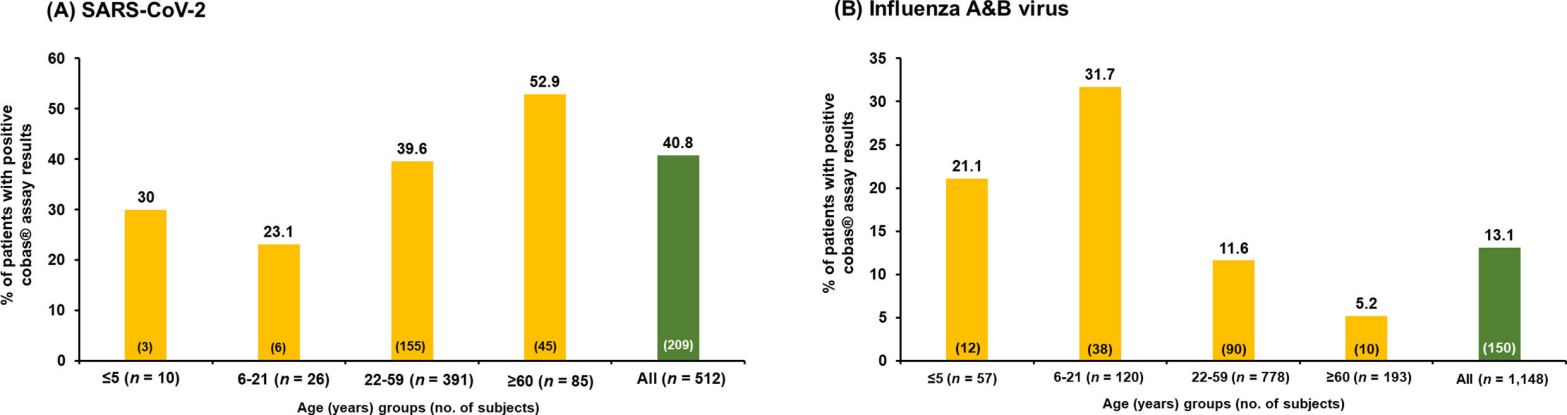
Percentages of patients with positive results for (**A**) SARS-CoV-2 and (**B**) influenza A and B viruses using cobas SARS-CoV-2 and influenza A/B qualitative assay by different age groups.

The prevalence of COVID-19, depicted in Fig. 4A, was stratified by age. Children and young adults (aged 6–21 years) exhibited the lowest prevalence at 23.1%, whereas adults aged ≥60 years had the highest prevalence at 52.9%. Across all evaluable subjects, the mean days from symptom onset was 2.6 days (range, 1–5 days). In the influenza cohort, those aged 6–21 years showed the highest prevalence at 31.7%, and adults aged ≥60 years had the lowest prevalence at 5.2% (Fig. 4B).

The sensitivity and specificity of the Panbio COVID-19/Flu A&B Panel for detecting SARS-CoV-2 are detailed in Table 2. Of the 512 evaluated subjects, 209 tested positive for COVID-19, whereas 303 tested negative using RT-PCR. The overall sensitivity of the Panbio COVID-19/Flu A&B Panel for COVID-19 was 80.4% (95% CI: 74.3, 85.5), and the specificity was 99.7% (95% CI: 98.2, 100.0). Notably, utilizing a Ct value of ≤35 as the cutoff, the sensitivity increased to 85.6% (95% CI: 79.8, 90.2), as outlined in Table 2.

**TABLE 2:** Performance of Panbio COVID-19/Flu A&B panel (Panbio assay) for the detection of SARS-CoV-2 by different age groups and regions of the patients compared with the results by cobas SARS-CoV-2 and influenza A/B qualitative assay (cobas assay)^*a*^

		Results by cobas assay for SARS-CoV-2			
Character	Performance of Panbio assay	Positive (Ct range: 13.95–38.20)	Negative	All	Cycle threshold values ≤35
All age groups	Positive	168	1	169	166
	Negative	41	302	343	28
	Total	209	303	512	194
	Sensitivity (95% CI) (%)			80.4 (74.3, 85.5)	85.6 (79.8, 90.2)
	Specificity (95% CI) (%)			99.7 (98.2, 100.0)	N/A
	Accuracy (95% CI) (%)			91.8 (89.1, 94.0)	
Age (≤13 years)	Positive	2	0	2	2
	Negative	1	11	12	1
	Total	3	11	14	3
	Sensitivity (95% CI) (%)			66.7 (9.4, 99.2)	66.7 (9.4, 99.2)
	Specificity (95% CI) (%)			100.0 (71.5, 100.0)	N/A
	Accuracy (95% CI) (%)			92.9 (66.1, 99.8)	
Age (14–23 years)	Positive	12	0	12	12
	Negative	1	34	35	1
	Total	13	34	47	13
	Sensitivity (95% CI) (%)			92.3 (64.0, 99.8)	92.3 (64.0, 99.8)
	Specificity (95% CI) (%)			100.0 (89.7, 100.0)	N/A
	Accuracy (95% CI) (%)			97.9 (88.7, 99.9)	
Age (24–64 years)	Positive	125	1	126	123
	Negative	31	226	257	23
	Total	156	227	383	146
	Sensitivity (95% CI) (%)			80.1 (73.0, 86.1)	84.2 (77.3, 89.7)
	Specificity (95% CI) (%)			99.6 (97.6, 100.0)	N/A
	Accuracy (95% CI) (%)			91.6 (88.4, 94.2)	
Age (≥65 years)	Positive	29	0	29	29
	Negative	8	31	39	3
	Total	37	31	68	32
	Sensitivity (95% CI) (%)			78.4 (61.8, 90.2)	90.6 (75.0, 98.0)
	Specificity (95% CI) (%)			100.0 (88.8, 100.0)	N/A
	Accuracy (95% CI) (%)			88.2 (78.1, 94.8)	
Age (<18 years)	Positive	2	0	2	2
	Negative	1	15	16	1
	Total	3	15	18	3
	Sensitivity (95% CI) (%)			66.7 (9.4, 99.2)	66.7 (9.4, 99.2)
	Specificity (95% CI) (%)			100.0 (78.2, 100.0)	N/A
	Accuracy (95% CI) (%)			94.4 (72.7, 99.9)	
Age (≥18 years)	Positive	166	1	167	164
	Negative	40	287	327	27
	Total	37	31	494	191
	Sensitivity (95% CI) (%)			80.6 (74.5, 85.8)	85.9 (80.1, 90.5)
	Specificity (95% CI) (%)			99.7 (98.1, 100.0)	N/A
	Accuracy (95% CI) (%)			91.7 (88.9, 94.)	
Region (APAC)	Positive	120	0	120	120
	Negative	20	71	91	14
	Total	140	71	211	134
	Sensitivity (95% CI) (%)			85.7 (78.8, 91.1)	89.6 (83.1, 94.2)
	Specificity (95% CI) (%)			100.0 (94.9, 100.0)	N/A
	Accuracy (95% CI) (%)			90.5 (85.7, 94.1)	
Region (Europe)	Positive	36	0	36	34
	Negative	13	126	139	8
	Total	49	126	175	42
	Sensitivity (95% CI) (%)			73.5 (58.9, 85.1)	81.0 (65.9, 91.4)
	Specificity (95% CI) (%)			100.0 (97.1, 100.0)	N/A
	Accuracy (95% CI) (%)			92.6 (87.6, 96.0)	
Region (USA)	Positive	12	1	13	12
	Negative	8	105	113	6
	Total	20	106	126	18
	Sensitivity (95% CI) (%)			60.0 (36.1, 80.9)	66.7 (41.0, 86.7)
	Specificity (95% CI) (%)			99.1 (94.9, 100.0)	N/A
	Accuracy (95% CI) (%)			92.9 (86.9, 96.7)	

^*a*^ CI, confidence interval; Ct, cycle threshold; N/A, not applicable.

Subgroup analyses were conducted on subjects categorized by age and regions, as outlined in Table 2. When stratified by age, individuals aged 24–64 years demonstrated a sensitivity of 80.1% (95% CI: 73.0, 86.1) and a specificity of 99.6% (95% CI: 97.6, 100.0). The sensitivity varied among other age groups: ≤13 [66.7% (95% CI: 9.4, 99.2)],14–23 [92.3% (95% CI: 64.0, 99.8)], and ≥65 [78.4% (95% CI: 61.8, 90.2)]. The corresponding specificities for these age ranges were 100.0% (95% CI: 71.5, 100.0), 100.0% (95% CI: 89.7, 100.0), and 100.0% (95% CI: 88.8, 100.0), respectively. When categorized by binary age range, the <18- and ≥18-year-old groups exhibited sensitivities of 66.7% (95% CI: 9.4, 99.2) and 80.6% (95% CI: 74.5, 85.8), and specificities of 100.0% (95% CI: 78.2, 100.0) and 99.7% (95% CI: 98.1, 100.0), respectively. Notably, the sensitivity of the <18-year-old group is based on only three subjects aged <13 years. For region-based analysis, sensitivities for APAC, EU, and the USA were 85.7% (95% CI: 78.8, 91.1), 73.5% (95% CI: 58.9, 85.1), and 60.0% (95% CI: 36.1, 80.9), with specificities of 100.0% (95% CI: 94.9, 100.0), 100.0% (95% CI: 97.1, 100.0), and 99.1% (95% CI: 94.9, 100.0), respectively.

The sensitivity and specificity of the Panbio COVID-19/Flu A&B Panel for detecting influenza are detailed in Table 3. Among the 1,148 evaluable subjects, 124 were positive for influenza A, and 1,024 were negative based on RT-PCR. The overall sensitivity for influenza A was 80.6% (95% CI: 72.6, 87.2), with a specificity of 99.3% (95% CI: 98.6, 99.7). For influenza B, 26 subjects were positive and 1,122 were negative, resulting in an overall sensitivity of 80.8% (95% CI: 60.6, 93.4) and a specificity of 99.4% (95% CI: 98.7, 99.7). The combined sensitivity for influenza A and B was 80.7% (95% CI: 73.4, 86.7), with a combined specificity of 98.6% (95% CI: 97.7, 99.2).

**TABLE 3:** Performance of Panbio COVID-19/Flu A&B panel (Panbio assay) for the detection of influenza A and B viruses by different age groups and regions of the patients compared with the results by cobas SARS-CoV-2 and influenza A/B qualitative assay (cobas assay)^*a*^

		Results by cobas assay for each indicated virus								
		Influenza A virus			Influenza B virus			Influenza A or B virus		
Character	Performance of Panbio assay	Positive	Negative	All	Positive	Negative	Total	Positive	Negative	All
All age groups	Positive	100	7	107	21	7	28	121	14	135
	Negative	24	1,017	1041	5	1,115	1120	29	984	1,013
	Total	124	1,024	1148	26	1,122	1148	150	998	1,148
	Sensitivity (95% CI) (%)			80.6 (72.6, 87.2)			80.8 (60.6, 93.4)			80.7 (73.4, 86.7)
	Specificity (95% CI) (%)			99.3 (98.6, 99.7)			99.4 (98.7, 99.7)			98.6 (97.7, 99.2)
	Accuracy (95% CI) (%)			97.3 (96.2, 98.2)			99.0 (98.2, 99.5)			96.3 (95.0, 97.3)
Age (≤13 years)	Positive	27	3	30	0	0	0	27	3	30
	Negative	7	78	85	0	115	115	7	78	85
	Total	34	81	115	0	115	115	34	81	115
	Sensitivity (95% CI) (%)			79.4 (62.1, 91.3)						79.4 (62.1, 91.3)
	Specificity (95% CI) (%)			96.3 (89.6, 99.2)			100.0 (96.8, 100.0)			96.3 (89.6, 99.2)
	Accuracy (95% CI) (%)			91.3 (84.6, 95.8)			100.0 (96.8, 100.0)			91.3 (84.6, 95.8)
Age (14–23 years	Positive	16	0	16	4	1	5	20	1	21
	Negative	2	97	99	0	110	110	2	92	94
	Total	18	97	115	4	111	115	22	93	115
	Sensitivity (95% CI) (%)			88.9 (65.3, 98.6)			100.0 (39.8, 100.0)			90.9 (70.8, 98.9)
	Specificity (95% CI) (%)			100.0 (96.3, 100.0)			99.1 (95.1, 100.0)			98.9 (94.2, 100.0)
	Accuracy (95% CI) (%)			98.3 (93.9, 99.8)			99.1 (95.3, 100.0)			97.4 (92.6, 99.5)
Age (24–64 years)	Positive	52	4	56	16	2	18	68	6	74
	Negative	13	708	721	5	754	759	18	685	703
	Total	65	712	777	21	756	777	86	691	777
	Sensitivity (95% CI) (%)			80.0 (68.2, 88.9)			76.2 (52.8, 91.8)			79.1 (69.0, 87.1)
	Specificity (95% CI) (%)			99.4 (98.6, 99.8)			99.7 (99.0, 100.0)			99.1 (98.1, 99.7)
	Accuracy (95% CI) (%)			97.8 (96.5, 98.7)			99.1 (98.2, 99.6)			96.9 (95.4, 98.0)
Age (≥65 years)	Positive	5	0	5	1	4	5	6	4	10
	Negative	2	134	136	0	136	136	2	129	131
	Total	7	134	141	1	140	141	8	133	141
	Sensitivity (95% CI) (%)			71.4 (29.0, 96.3)			100.0 (2.5, 100.0)			75.0 (34.9, 96.8)
	Specificity (95% CI) (%)			100.0 (97.3, 100.0)			97.1 (92.8, 99.2)			97.0 (92.5, 99.2)
	Accuracy (95% CI) (%)			98.6 (95.0, 99.8)			97.2 (92.9, 99.2)			95.7 (91.0, 98.4)
Age (<18 years)	Positive	34	3	37	0	0	0	34	3	37
	Negative	8	87	95	0	132	132	8	87	95
	Total	42	90	132	0	132	132	42	90	132
	Sensitivity (95% CI) (%)			81.0 (65.9, 91.4)						81.0 (65.9, 91.4)
	Specificity (95% CI) (%)			96.7 (90.6, 99.3)			100.0 (97.2, 100.0)			96.7 (90.6, 99.3)
	Accuracy (95% CI) (%)			91.7 (85.6, 95.8)			100.0 (97.2, 100.0)			91.7 (85.6, 95.8)
Age (≥18 years)	Positive	66	4	70	21	7	28	87	11	98
	Negative	16	930	946	5	983	988	21	897	918
	Total	82	934	1,016	26	990	1,016	108	908	1,016
	Sensitivity (95% CI) (%)			80.5 (70.3, 88.4)			80.8 (60.6, 93.4)			80.6 (71.8, 87.5)
	Specificity (95% CI) (%)			99.6 (98.9, 99.9)			99.3 (98.5, 99.7)			98.8 (97.8, 99.4)
	Accuracy (95% CI) (%)			98.0 (97.0, 98.8)			98.8 (97.9, 99.4)			96.9 (95.6, 97.8)
Region (Asia)	Positive	81	0	81	2	2	4	83	2	85
	Negative	20	375	395	1	471	472	21	370	391
	Total	101	375	476	3	473	476	104	372	476
	Sensitivity (95% CI) (%)			80.2 (71.1, 87.5)			66.7 (9.4, 99.2)			79.8 (70.8, 87.0)
	Specificity (95% CI) (%)			100.0 (99.0, 100.0)			99.6 (98.5, 99.9)			99.5 (98.1, 99.9)
	Accuracy (95% CI) (%)			95.8 (93.6, 97.4)			99.4 (98.2, 99.9)			95.2 (92.8, 96.9)
Region (Europe)	Positive	16	4	20	19	5	24	35	9	44
	Negative	3	492	495	4	487	491	7	464	471
	Total	19	496	515	23	492	515	42	473	515
	Sensitivity (95% CI) (%)			84.2 (60.4, 96.6)			82.6 (61.2, 95.0)			83.3 (68.6, 93.0)
	Specificity (95% CI) (%)			99.2 (97.9, 99.8)			99.0 (97.6, 99.7)			98.1 (96.4, 99.1)
	Accuracy (95% CI) (%)			98.6 (97.2, 99.5)			98.2 (96.7, 99.2)			96.9 (95.0, 98.2)
Region (USA)	Positive	3	3	6	0	0	0	3	3	6
	Negative	1	150	151	0	157	157	1	150	151
	Total	4	153	157	0	157	157	4	153	157
	Sensitivity (95% CI) (%)			75.0 (19.4, 99.4)						75.0 (19.4, 99.4)
	Specificity (95% CI) (%)			98.0 (94.4, 99.6)			100.0 (97.7, 100.0)			98.0 (94.4, 99.6)
	Accuracy (95% CI) (%)			97.5 (93.6, 99.3)			100.0 (97.7, 100.0)			97.5 (93.6, 99.3)
	Specificity (95% CI) (%)			99.4 (98.7, 99.8)			99.3 (98.5, 99.7)			98.6 (97.6, 99.3)
	Accuracy (95% CI) (%)			97.3 (96.1, 98.2)			98.8 (97.9, 99.4)			96.1 (94.7, 97.2)

^*a*^ CI, confidence interval; Ct, cycle threshold.

Subgroup analyses were conducted based on age, and regions as outlined in Table 3. When stratified by age range, the observed sensitivity for influenza A was 79.4% (95% CI: 62.1, 91.3), 88.9% (95% CI: 65.3, 98.6), 80.0% (95% CI: 68.2, 88.9), and 71.4% (95% CI: 29.0, 96.3) for the age range of ≤13, 14–23, 24–64, and ≥65 years, respectively. For influenza B, the observed sensitivity was 100.0% (95% CI: 39.8, 100.0), 76.2% (95% CI: 52.8, 91.8), and 100.0% (95% CI: 2.5, 100.0) for the age range of 14–23, 24–64, and ≥65 years, respectively. No influenza B-positive subjects were detected in the ≤13-year-old age range. Specificities for influenza A range from 96.3% to 100.0%, and for influenza B, from 97.1% to 100%, across different age groups. When categorized by binary age range, the observed sensitivity for influenza A was 81.0% (95% CI: 65.9, 91.4) and 80.5% (95% CI: 70.3, 88.4) for the <18 and ≥18-year-old age range, respectively. For influenza B, the sensitivity was 80.8% (95% CI: 60.6, 93.4) for the ≥18-year-old age range, and no influenza B-positive cases were detected in the <18-year-old group. Specificities for influenza A and B ranged from 96.7% to 100%. Regarding region-based analysis, the observed sensitivities for influenza A were 80.2% (95% CI: 71.1, 87.5), 84.2% (95% CI: 60.4, 96.6), and 75.0% (95% CI: 19.4, 99.4) for APAC, EU, and the USA, respectively. For influenza B, the observed sensitivities were 66.7% (95% CI: 9.4, 99.2) and 82.6% (95% CI: 61.2, 95.0) for Asia and Europe, respectively. No influenza B-positive cases were found in the USA. The specificities for both flu types varied from 98.0% to 100% across different regions.

**Fig 5:**
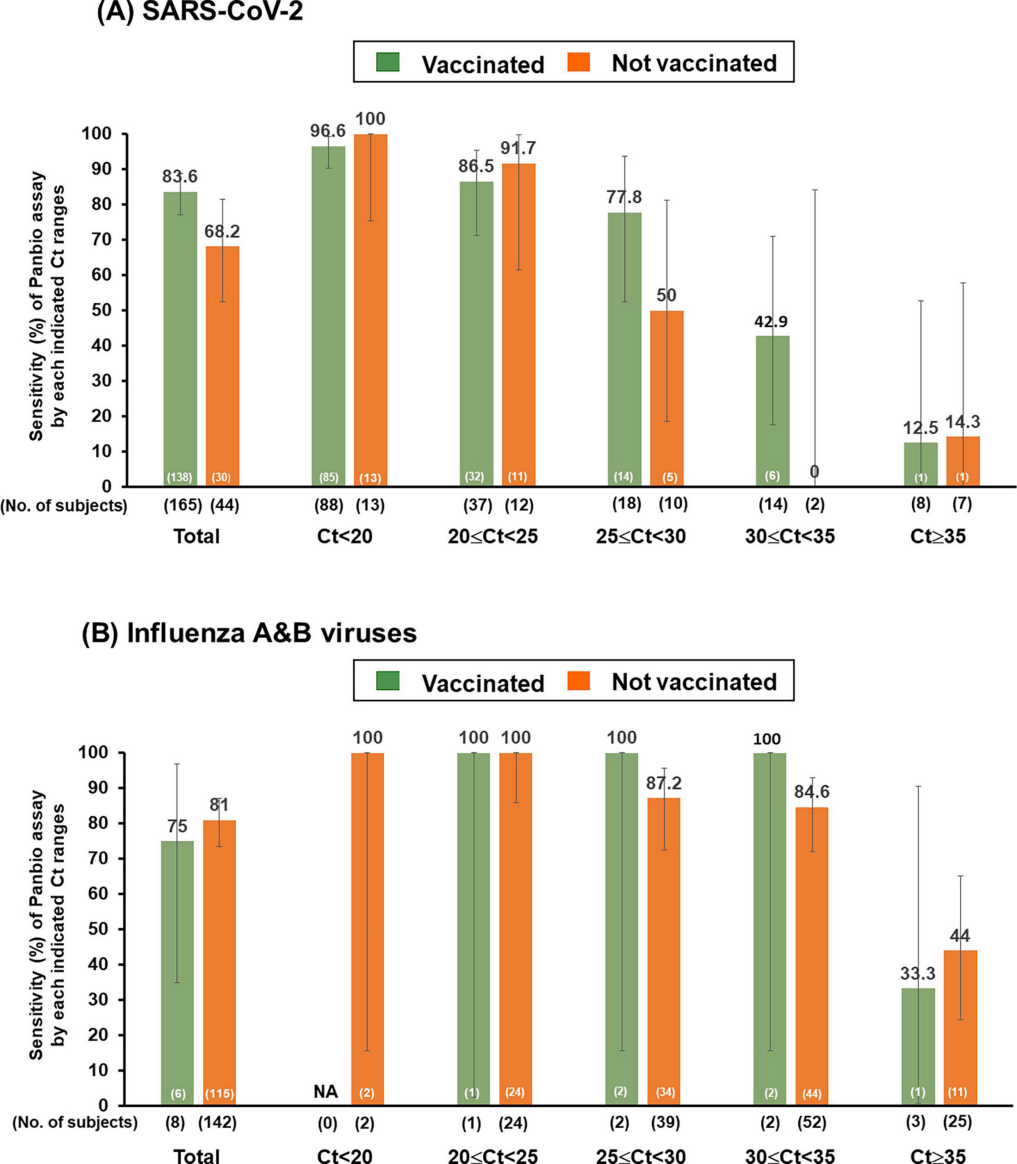
Sensitivity of Panbio COVID-19/Flu A&B panel assay (Panbio assay) for detecting (**A**) SARS-CoV-2 and (**B**) influenza A and B viruses according to vaccination status generated by cobas SARS-CoV-2 and influenza A/B qualitative assay.

Regarding vaccination status in the COVID-19 cohort, the observed overall sensitivities for the vaccinated and non-vaccinated groups were 83.6% (95% CI: 77.1, 88.9) and 68.2% (95% CI: 52.4, 81.4), respectively. The observed sensitivities for influenza A and B were 75.0% (95% CI: 34.9, 96.8) and 81.0% (95% CI: 73.6, 87.1) across vaccinated and non-vaccinated groups, respectively (Fig. 5A and B; Tables S1 and S2). In the COVID-19 cohort, stratifying by Ct values showed varying sensitivities. For the vaccinated group, sensitivities ranged from 12.5% (Ct ≥35) to 96.6% (Ct <20). The non-vaccinated group had sensitivities ranging from 14.3% (Ct ≥35) to 100% (Ct <20) (Fig. 5A; Table S1). In the influenza cohort, the sensitivities for vaccinated individuals in different Ct ranges were diverse, ranging from 33.3% (Ct ≥35) to 100% (20 ≤ Ct < 35). Non-vaccinated individuals showed sensitivities ranging from 44% (Ct ≥35) to 100% (Ct <25) (Fig. 5B; Table S2).

**Fig 6:**
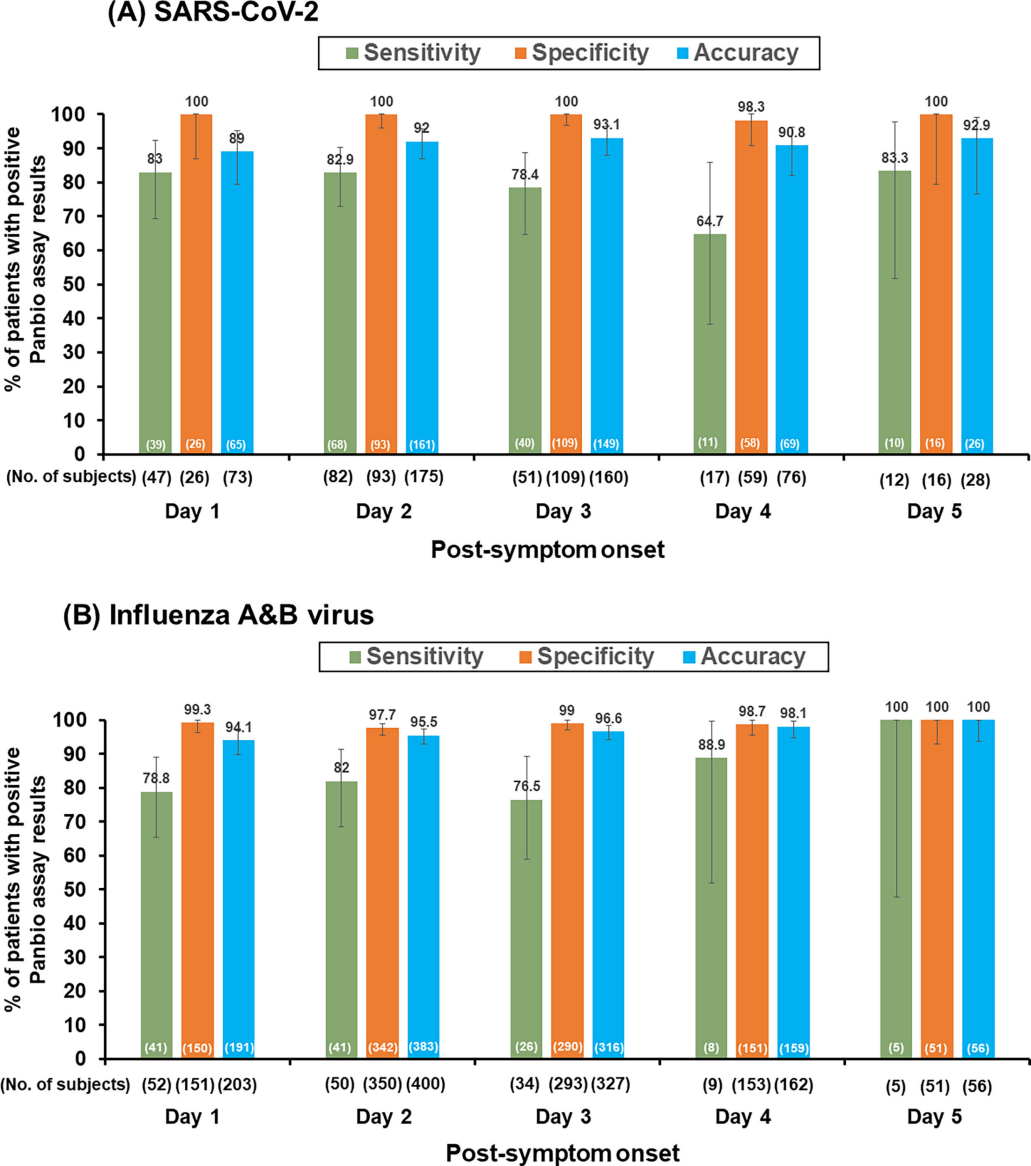
Performance of Panbio COVID-19/Flu A&B panel assay (Panbio assay) for (**A**) SARS-CoV-2 and (**B**) combined influenza A and influenza B by post-symptom days (Days 1– 5).

In the COVID-19 cohort, sensitivity and specificity were further stratified by days post-onset of symptoms (Fig. 6A), revealing the lowest sensitivity observed at Day 4. In the influenza cohort, sensitivity and specificity stratified by days post-symptom onset for influenza A/B are demonstrated in Fig. 6B, with the lowest sensitivity at Day 3.

**Fig 7:**
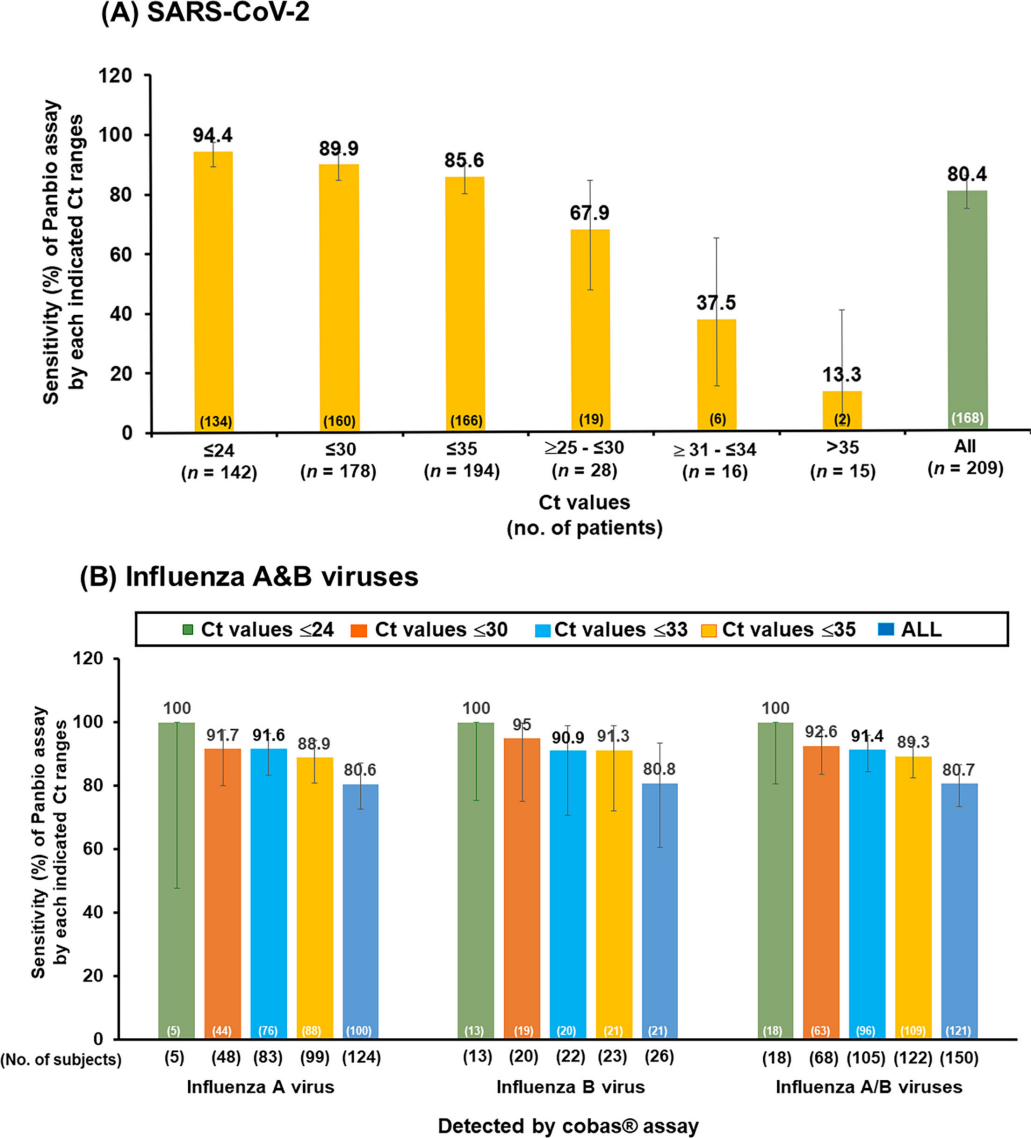
Sensitivity of Panbio COVID-19/Flu A&B Panel assay (Panbio assay) for detecting (**A**) SARS-CoV-2 and (**B**) influenza A and B viruses according to the cycle threshold (Ct) values generated by cobas SARS-CoV-2 & influenza A/B qualitative assay.

Within the COVID-19 cohort, sensitivity was stratified by Ct value (Ct ≤24, Ct ≤30, and Ct ≤35). A notable increase in sensitivity was observed at lower Ct values, indicating higher viral loads. When the sensitivity was further stratified by Ct values within defined ranges (25 ≤ Ct ≤ 30, 30 < Ct ≤ 35, and Ct >35), an increase in sensitivity was observed within lower Ct value ranges (Fig. 7A). In the influenza cohort, when the sensitivity was stratified by Ct value for influenza A/B, the observed values were 89.3% (95% CI: 82.5, 94.2), 91.4% (95%CI: 84.4, 96.0), 92.6% (95% CI: 83.7, 97.6), and 100.0% (95% CI: 81.5, 100.0) for Ct values ≤ 35, ≤33, ≤30, and ≤24, respectively. This demonstrates a marked increase in sensitivity as Ct values decrease, indicating higher viral loads (Fig. 7B).

From a safety evaluation standpoint, this study reported three non-serious unrelated adverse events, which were all mild epistaxis that was resolved. No serious adverse events (SAEs) were reported in this study.

## DISCUSSION

This international, prospective, multi-center study illustrates the real-world clinical performance of the Panbio COVID-19/Flu A&B Panel, exhibiting sensitivities exceeding 80.0% and specificities surpassing 98.0% for all three viruses evaluated. As we move towards the endemic phase of the COVID-19 disease, the requirement for differential diagnosis of COVID-19 and influenza A and B becomes increasingly crucial. Patients infected with SARS-CoV-2 can present with symptoms akin to influenza, including fever, chills, cough, fatigue, and myalgia (20), posing challenges in their differential diagnosis based solely on symptoms. However, the treatment and duration of isolation for each illness differ significantly (21). Lateral flow testing is mostly used for infectivity diagnosis and monitoring. With the likelihood of co-circulation of SARS-CoV-2 and influenza viruses (22, 23), the rapid differential detection of all three targets using a multiplex rapid antigen test kit could help provide the proper patient management and treatment.

The continual emergence and global spread of new SARS-CoV-2 strains persistently challenge diagnostic tests due to viral diversity (24). The SARS-CoV-2 target used by this multiplex kit is the nucleocapsid protein, which has been shown to be highly conserved among different SARS-CoV-2 variants (25). Notably, this large multi-center study was conducted during the most recent respiratory season (from December 2022 to March 2023), encompassing testing for the currently circulating COVID-19 and flu strains. Furthermore, a recent prospective study demonstrated the high accuracy in detecting the prevalent omicron variant of a prior generation device (26). The device evaluated in that particular study shares the same combination and concentration of COVID-19 antibodies directed against SAR-CoV-2 nucleoproteins as the device used in the current study, and therefore, both devices have the same sensitivity for COVID-19 variants. The current study also used prospectively collected fresh samples and offered several advantages over prior studies reliant on frozen samples. The use of fresh samples provides a more realistic representation of the real-world situation, offering valuable insights into the dynamic nature of these critical infections. The advantage ranges from maintaining biological relevance to capturing the dynamic interplay between the virus and the host, ultimately improving the quality and applicability of the research findings for public health and clinical practice. The use of fresh samples is also in keeping with the intended use of the Panbio COVID-19/Flu A&B Panel, which is the qualitative detection of nucleocapsid proteins from SARS-CoV-2 and nucleoprotein influenza A and B antigens in human NMT swab specimens from symptomatic individuals. Additionally, fresh samples were used to address the EU Common Specification (EU) 2022/1107 – Annex XIII (for devices intended for detection or qualification of markers of SARS-CoV-2 infection), which requires a minimum of 100 SARS-CoV-2 reference-positive subjects and a minimum of 300 SARS-CoV-2 reference-negative subjects to be prospectively enrolled in multiple clinical sites to detect predominant variants in circulation for COVID-19 (27).

The present study was the largest to date using fresh samples to evaluate the performance of a single-strip COVID-19 and influenza combination lateral flow test. In the COVID-19 cohort, the Panbio COVID-19/Flu A&B Panel exhibited an overall sensitivity of 80.4% (95% CI: 74.3, 85.5) and specificity of 99.7% (95% CI: 98.2, 100.0) for COVID-19. The performance acceptance criteria for COVID-19 as defined in the study protocol were a sensitivity of ≥80% and a specificity of ≥98% when compared with RT-PCR per the EU Common Specification IVDR requirement (28). Although approximately 20% of cases may be at risk of being missed by the Panbio COVID-19/Flu A&B panel, particularly in patients aged <18 and ≥65 years, the protocol acceptance criteria for COVID-19 have been met. The sensitivity of minors (<18 years) (66.7%) and adults (≥18 years) (80.6%) was not significantly different (*P* = 0.4824, Fisher’s exact test). Of note, the sensitivity of the <18-year-old group was based on the three subjects aged <13 years. In addition, based on a literature review of rapid influenza diagnostic tests that detect influenza A and B nucleoprotein antigens in respiratory secretions, their sensitivity is low to moderate, and ranges from 50% to 80% (20, 29). These meta-analyses are based on clinical performance data from >150 studies. One meta-analysis of observational studies of rapid influenza antigen tests revealed a moderate sensitivity of 62% (30). Another meta-analysis reported pooled sensitivities of 54% and 53% to detect influenza A and B virus antigens, respectively, compared with RT-PCR (31). The influenza sensitivity range of 50%–80% that is reported in these independent meta-analyses covering >150 studies is therefore deemed the appropriate benchmark for influenza sensitivity.

When the data were stratified by Ct value, the sensitivity of the device correlates with the viral loads detected by the reference RT-PCR method (based on Ct values). For COVID-19 cases with a Ct ≤35, the sensitivity was 85.6%. With Ct of ≤30, the sensitivity increased to 89.9%. This indicated that the Panbio COVID-19/Flu A&B Panel can maintain stable testing accuracy. Compared with RT-PCR, the Panbio COVID-19/Flu A&B Panel offers advantages, such as the ability to conduct multiple repeat tests, rapid execution, and lower costs, making it more feasible for epidemic prevention activities. Minor differences in sensitivity of the device were observed when the data were stratified by region. This is likely due to the local prevalence rates of SARS-CoV-2 in the different regions in APAC, EU, and the USA. Furthermore, the collection time point (days since symptom onset) did not affect the sensitivity of the device.

Regarding influenza A, the overall sensitivity and specificity of the Panbio COVID-19/Flu A&B Panel was 80.6% (95% CI: 72.6, 87.2) and 99.3% (95% CI: 98.6, 99.7), respectively. Sensitivity for influenza A correlated with viral load, demonstrating a sensitivity of >90% for samples with Ct values ≤30. Similar to COVID-19, the collection time point (days since symptom onset) and vaccine status had no impact on the sensitivity of the device. The Panbio COVID-19/Flu A&B Panel performs equally well in detecting influenza A in both minors and adults, with sensitivities >80%.

For influenza B, the overall sensitivity and specificity of the Panbio COVID-19/Flu A&B Panel were 80.8% (95% CI: 60.6, 93.4) and 99.4% (95% CI: 98.7, 99.7), respectively. Sensitivity to influenza B also correlated with Ct values, showing higher sensitivity for samples with higher viral loads. However, due to the low prevalence of influenza B across APAC, EU and the USA, the performance results for influenza B sensitivity were estimated with a wide confidence interval.

Notably, this assay achieved a sensitivity of 94.4% (95% CI: 89.2, 97.5) in samples with Ct ≤24 for COVID-19 and 92.6% (95% CI: 83.7, 97.6) in samples with Ct ≤30 for influenza A/B combined. Although the cobas assay is a qualitative assay, the correlation of Ct values with viral load, particularly emphasizing Ct values between 25 and 30 as thresholds beyond which viral isolation becomes less likely, has been well-established for COVID-19 (32, 33). A previous study also demonstrated that the infectivity (as defined by growth in cell culture) was significantly reduced when Ct values >24 (34). If validated, the Ct proxy value could provide a streamlined and less labor-intensive metric for bedside clinicians and infection control practitioners. This would facilitate efficient assessment of whether a patient has an active, contagious infection, aiding in prompt decisions regarding necessary treatment or isolation (35, 36).

A previous report indicated that the diagnostic sensitivity of SARS-CoV-2 rapid antigen testing can be influenced by vaccination status (37). In this study, we found that the vaccine status did not impact the sensitivity of the device, signifying a significant validation of test efficacy of the Panbio COVID-19/Flu A&B Panel in vaccinated individuals. Our study showcased a high sensitivity of 83.6% (95% CI: 77.1, 88.9) in vaccinated patients, comparable to or even exceeding results from previous studies (38–40). Notably, the test exhibited similar sensitivity in both vaccinated and non-vaccinated patients. Prior studies have suggested diminished diagnostic sensitivity in individuals with breakthrough COVID-19 infection post-vaccination (39, 40), likely due to the generally lower viral load associated with vaccine breakthrough infections (37). Unlike other studies, our findings indicate comparable sensitivity between the two groups, possibly attributable to the comparable Ct values between the two groups.

Furthermore, our investigation examined the impact of influenza vaccination on the sensitivity of influenza diagnostics. Despite the well-established effect of live, attenuated influenza vaccine (e.g., FluMist) on rapid antigen test results for influenza detection (41, 42), little is known about the influence of inactivated influenza vaccinations on diagnostic sensitivity. Our study revealed that the high sensitivity observed in non-vaccinated patients was maintained in vaccinated patients. These results suggest that the Panbio COVID-19/Flu A&B Panel is a reliable tool for detecting breakthrough infections of COVID-19 or influenza.

Despite encompassing a substantial number of subjects, this study has some limitations. Primarily, the low prevalence of influenza B during the study period resulted in a markedly lower positive rate for influenza B (2.3%) than influenza A (10.8%). No influenza B was detected in the USA, and only a limited number of influenza B-positive subjects were enrolled in APAC. Moreover, no influenza B-positive minor subjects (<18 years old) were recruited, rendering the sample size insufficient for age-based stratification, assessment of vaccination status, and analyses based on days since symptom onset. Given the scarce prevalence of influenza B across APAC, Europe, and the USA during the study period, the performance results for influenza B sensitivity have been estimated with a wide confidence interval. Second, the detailed vaccination information was lacking. Different vaccines were used across the different sites, and the dose and time elapsed since vaccination may influence the viral load of the disease, potentially impacting the sensitivity of the kit due to lower viral load. Third, the collection of samples for the Panbio COVID-19/Flu A&B Panel from the NMT differed from the NP area sampling for the cobas assay. This discrepancy in sample areas could potentially lead to variations in viral loads. NP samples are considered the gold standard for COVID-19 diagnostics per IVDR (28), and were therefore used for the reference testing to provide the most stringent sensitivity comparison to the Panbio COVID-19/Flu A&B Panel device. Fourth, studies were carried out in various geographical locations that encountered COVID-19 outbreaks at different periods and potentially with different subvariants, with disparate schedules for vaccine rollouts during the pandemic. Finally, the requirement for some sites to freeze samples for transport in RT-PCR assays introduces a potential variability between frozen and fresh samples.

The strengths of this study include a large, multi-center, ethnically diverse group of participants in both COVID-19 and influenza A/B cohorts. This feature reflects the heterogeneity of actual users in real-world scenarios, providing a more accurate estimation of sensitivity and specificity. Additionally, the inclusion of multiple sites utilizing fresh samples underscores the practical applicability of the device in everyday situations, distinguishing it from prior studies that predominantly relied on frozen or stored samples (43, 44). Furthermore, we conducted sensitivity analyses, stratifying the results based on various criteria, such as age, region, Ct value, and vaccination status, thereby offering additional insights into the performance of the device.

In conclusion, based on the clinical performance data obtained from this study, the Panbio COVID-19/Flu A&B Panel demonstrates suitability as a rapid test for detecting COVID-19 and influenza in symptomatic subjects in diverse populations globally, exhibiting high sensitivity.

## Data Availability

The data that support the findings of this study are available from the corresponding author, P.-R.H., upon reasonable request.

## SUPPLEMENTAL MATERIAL

Supplemental tables jcm.00207-24-s0001.docx Tables S1 and S2.

ASM does not own the copyrights to Supplemental Material that may be linked to, or accessed through, an article. The authors have granted ASM a non-exclusive, world-wide license to publish the Supplemental Material files. Please contact the corresponding author directly for reuse.
