# Supplementary material for: Performance evaluation of the Panbio COVID-19/Flu A&B Panel for detection of SARS-CoV-2, influenza A, and influenza B antigens using mid-turbinate nasal swabs
Source: J Clin Microbiol. 2024 Jun 18;62(7):e00207-24. doi: 10.1128/jcm.00207-24 (PMC11250729; doi:10.1128/jcm.00207-24)
Supplement: Supplemental tables — Tables S1 and S2. [file jcm.00207-24-s0001.docx]

# Supplementary Table 1: Performance of Panbio™ COVID-19/Flu A&B Panel (Panbio assay) for the detection of SARS-CoV-2 stratified by COVID-19 vaccination status of the patients as compared with the results by cobas® SARS-CoV-2 & Influenza A/B qualitative assay (cobas® assay).

|  | | **Results by cobas® assay for SARS-CoV-2** | | | | | | | | |
| --- | --- | --- | --- | --- | --- | --- | --- | --- | --- | --- |
|  |  | **All subjects** | | | **Vaccinated subjects** | | | **Non-vaccinated subjects** | | |
| **Ranges of Ct values** | **Performance of**  **Panbio assay** | **Positive** | **Negative** | **Total** | **Positive** | **Negative** | **Total** | **Positive** | **Negative** | **Total** |
| All | Positive | 168 | 1 | 169 | 138 | 1 | 139 | 30 | 0 | 30 |
|  | Negative | 41 | 302 | 343 | 27 | 195 | 222 | 14 | 107 | 121 |
|  | Total | 209 | 303 | 512 | 165 | 196 | 361 | 44 | 107 | 151 |
|  | Sensitivity  (95% CI) (%) |  |  | 80.4  (74.3, 85.5) |  |  | 83.6  (77.1, 88.9) |  |  | 68.2  (52.4, 81.4) |
|  | Specificity  (95% CI) (%) |  |  | 99.7  (98.2, 100.0) |  |  | 99.5  (97.2, 100.0) |  |  | 100.0  (96.6, 100.0) |
|  | Accuracy  (95% CI) (%) |  |  | 91.8  (89.1, 94.0) |  |  | 92.2  (89.0, 94.8) |  |  | 90.7  (84.9, 94.8) |
| Ct < 20 | Positive | 98 |  |  | 85 |  |  | 13 |  |  |
|  | Negative | 3 |  |  | 3 |  |  | 0 |  |  |
|  | Total | 101 |  |  | 88 |  |  | 13 |  |  |
|  | Sensitivity  (95% CI) (%) | 97.0 (91.6, 99.4) | | | 96.6 (90.4, 99.3) | | | 100.0 (75.3, 100.0) | | |
|  | Specificity  (95% CI) (%) | N/A | | | N/A | | | N/A | | |
| 20 ≤ Ct < 25 | Positive | 43 |  |  | 32 |  |  | 11 |  |  |
|  | Negative | 6 |  |  | 5 |  |  | 1 |  |  |
|  | Total | 49 |  |  | 37 |  |  | 12 |  |  |
|  | Sensitivity  (95% CI) (%) | 87.8 (75.2, 95.4) | | | 86.5 (71.2, 95.5) | | | 91.7 (61.5, 99.8) | | |
|  | Specificity  (95% CI) (%) | N/A | | | N/A | | | N/A | | |
| 25 ≤ Ct < 30 | Positive | 19 |  |  | 14 |  |  | 5 |  |  |
|  | Negative | 9 |  |  | 4 |  |  | 5 |  |  |
|  | Total | 28 |  |  | 18 |  |  | 10 |  |  |
|  | Sensitivity  (95% CI) (%) | 67.9 (47.6, 84.1) | | | 77.8 (52.4, 93.6) | | | 50.0 (18.7, 81.3) | | |
|  | Specificity  (95% CI) (%) | N/A | | | N/A | | | N/A | | |
| 30 ≤ Ct < 35 | Positive | 6 |  |  | 6 |  |  | 0 |  |  |
|  | Negative | 10 |  |  | 8 |  |  | 2 |  |  |
|  | Total | 16 |  |  | 14 |  |  | 2 |  |  |
|  | Sensitivity  (95% CI) (%) | 37.5 (15.2, 64.6) | | | 42.9 (17.7, 71.1) | | | 0.0 (0.0, 84.2) | | |
|  | Specificity  (95% CI) (%) | N/A | | | N/A | | | N/A | | |
| Ct ≥ 35 | Positive | 2 |  |  | 1 |  |  | 1 |  |  |
|  | Negative | 13 |  |  | 7 |  |  | 6 |  |  |
|  | Total | 15 |  |  | 8 |  |  | 7 |  |  |
|  | Sensitivity  (95% CI) (%) | 13.3 (1.7, 40.5) | | | 12.5 (0.3, 52.7) | | | 14.3 (0.4, 57.9) | | |
|  | Specificity  (95% CI) (%) | N/A | | | N/A | | | N/A | | |

CI, confidence interval; Ct, Cycle threshold; N/A, not applicable

# Supplementary Table 2: Performance of Panbio™ COVID-19/Flu A&B Panel (Panbio assay) for the detection of influenza A & B viruses by influenza vaccination status of the patients as compared with the results by cobas® SARS-CoV-2 & Influenza A/B qualitative assay (cobas® assay).

|  | | **Results by cobas® assay for influenza A&B viruses** | | | | | | | | |
| --- | --- | --- | --- | --- | --- | --- | --- | --- | --- | --- |
|  |  | **All subjects** | | | **Vaccinated subjects** | | | **Non-vaccinated subjects** | | |
| **Ranges of Ct values** | **Performance of**  **Panbio assay** | **Positive** | **Negative** | **Total** | **Positive** | **Negative** | **Total** | **Positive** | **Negative** | **Total** |
| All | Positive | 121 | 14 | 135 | 6 | 2 | 8 | 115 | 12 | 127 |
|  | Negative | 29 | 984 | 1013 | 2 | 140 | 142 | 27 | 844 | 871 |
|  | Total | 150 | 998 | 1148 | 8 | 142 | 150 | 142 | 856 | 998 |
|  | Sensitivity  (95% CI) (%) |  |  | 80.7  (73.4, 86.7) |  |  | 75.0  (34.9, 96.8) |  |  | 81.0  (73.6, 87.1) |
|  | Specificity  (95% CI) (%) |  |  | 98.6  (97.7, 99.2) |  |  | 98.6  (95.0, 99.8) |  |  | 98.6  (97.6, 99.3) |
|  | Accuracy  (95% CI) (%) |  |  | 96.3  (95.0, 97.3) |  |  | 97.3  (93.3, 99.3) |  |  | 96.1  (94.7, 97.2) |
| Ct < 20 | Positive | 2 |  |  | 0 |  |  | 2 |  |  |
|  | Negative | 0 |  |  | 0 |  |  | 0 |  |  |
|  | Total | 2 |  |  | 0 |  |  | 2 |  |  |
|  | Sensitivity  (95% CI) (%) | 100.0 (15.8, 100.0) | | | N/A | | | 100.0 (15.8, 100.0) | | |
|  | Specificity  (95% CI) (%) | N/A | | | N/A | | | N/A | | |
| 20 ≤ Ct < 25 | Positive | 25 |  |  | 1 |  |  | 24 |  |  |
|  | Negative | 0 |  |  | 0 |  |  | 0 |  |  |
|  | Total | 25 |  |  | 1 |  |  | 24 |  |  |
|  | Sensitivity  (95% CI) (%) | 100.0 (86.3, 100.0) | | | 100.0 (2.5, 100.0) | | | 100.0 (85.8, 100.0) | | |
|  | Specificity  (95% CI) (%) | N/A | | | N/A | | | N/A | | |
| 25 ≤ Ct < 30 | Positive | 36 |  |  | 2 |  |  | 34 |  |  |
|  | Negative | 5 |  |  | 0 |  |  | 5 |  |  |
|  | Total | 41 |  |  | 2 |  |  | 39 |  |  |
|  | Sensitivity  (95% CI) (%) | 87.8 (73.8, 95.9) | | | 100.0 (15.8, 100.0) | | | 87.2 (72.6, 95.7) | | |
|  | Specificity  (95% CI) (%) | N/A | | | N/A | | | N/A | | |
| 30 ≤ Ct < 35 | Positive | 46 |  |  | 2 |  |  | 44 |  |  |
|  | Negative | 8 |  |  | 0 |  |  | 8 |  |  |
|  | Total | 54 |  |  | 2 |  |  | 52 |  |  |
|  | Sensitivity  (95% CI) (%) | 85.2 (72.9, 93.4) | | | 100.0 (15.8, 100.0) | | | 84.6 (71.9, 93.1) | | |
|  | Specificity  (95% CI) (%) | N/A | | | N/A | | | N/A | | |
| Ct ≥ 35 | Positive | 12 |  |  | 1 |  |  | 11 |  |  |
|  | Negative | 16 |  |  | 2 |  |  | 14 |  |  |
|  | Total | 28 |  |  | 3 |  |  | 25 |  |  |
|  | Sensitivity  (95% CI) (%) | 42.9 (24.5, 62.8) | | | 33.3 (0.8, 90.6) | | | 44.0 (24.4, 65.1) | | |
|  | Specificity  (95% CI) (%) | N/A | | | N/A | | | N/A | | |

CI, confidence interval; Ct, Cycle threshold; N/A, not applicable
